# Improving mental health through neighbourhood regeneration: the role of cohesion, belonging, quality and disorder

**DOI:** 10.1093/eurpub/ckz221

**Published:** 2019-12-10

**Authors:** Giles Greene, David Fone, Daniel Farewell, Sarah Rodgers, Shantini Paranjothy, Bethan Carter, James White

**Affiliations:** 1 Division of Population Medicine, School of Medicine, Neuadd Meirionnydd, Cardiff University, Heath Park, Cardiff, UK; 2 Institute of Population Health Sciences, University of Liverpool, Liverpool, UK; 3 Centre for Trials Research, School of Medicine, Cardiff University, Cardiff, UK; 4 Centre for the Development and Evaluation of Complex Interventions for Public Health Improvement (DECIPHer), College of Biomedical and Life Sciences, Cardiff University, Cardiff, UK

## Abstract

Poor mental health has been associated with socioeconomic deprivation. The aim was to describe possible mechanisms underpinning the narrowing of mental health inequalities demonstrated by Communities First, an area-wide regeneration programme in Wales, UK. Propensity score matched data from the Caerphilly Health and Social Needs Electronic Cohort Study, assessed changes in mental health, neighbourhood-level social cohesion, belongingness, quality and disorder. A multiple mediation analysis found c.76% of the total indirect effect was accounted for by neighbourhood quality and disorder. Targeted regeneration that increases neighbourhood quality and reduced neighbourhood disorder could mitigate the mental health inequalities associated with socioeconomic deprivation.

## Introduction

In the UK, the Marmot Review of Health Inequalities and US National Institute on Minority Health and Health Disparities recommended community regeneration programmes are implemented to reduce health inequalities.^1^ Caerphilly, Wales is a post-industrial area with historic social and health inequalities reporting mental health scores significantly below the Welsh average. A Welsh Government implemented regeneration programme ‘Communities First’, delivered to the 10% most deprived neighbourhoods in Wales (UK) was associated with a small improvement in the mental health of Communities First residents compared with propensity matched control group residents; hence inequalities narrowed.^2^ Little is known of the mechanisms explaining this association. We examined the role of three factors in mediating the effect of Community First: social cohesion, neighbourhood belonging and neighbourhood quality and disorder.

## Methods

### Participants

Data are from the Caerphilly Health and Social Needs Electronic Cohort Study, a prospective cohort study of adult residents of Caerphilly County Borough (CCB), Wales, UK.^2^ Briefly, a baseline postal questionnaire in 2001 elicited 10 892 responses (60.6%). In 2008, a follow-up survey on the 9551 participants still residing in the borough provided 4426 valid mental health scores.

### Intervention

‘Communities First’ was an area-wide regeneration programme delivered in the 10% most deprived of the 1896 lower super output areas (LSOAs; average population 1630) in Wales determined by the 2000 Welsh Index of Multiple Deprivation.^3^ In 2001, each local authority with Communities First areas established multi-agency partnership boards to identify regeneration opportunities and apply to potential funders. Data provided by CCB Council identified the LSOAs receiving Communities First funding (intervention areas) and those that did not (control areas).^2^ Funding included activities addressing: (i) crime; (ii) education; (iii) health; (iv) housing and physical environment; (v) vocational training and business support; and (vi) community. More detail is provided elsewhere.^2^ The 110 LSOAs within CCB were categorized into 35 interventions and 75 control LSOAs.

### Outcome measure

Mental health was assessed using the 5-item Mental Health Inventory (MHI-5), a subscale of the Short Form-36 version 2. The MHI-5 is a well validated measure of mental health in the general population and is effective in screening mood and anxiety disorders using Diagnostic Interview Schedules.^4^ Changes in mental health were calculated by subtracting Wave 1 from Wave 2 scores, positive values indicated an improvement in mental health.

### Neighbourhood mediators

In 2001 and 2008, an assessment of social cohesion, neighbourhood belonging, quality and disorder was made.^5^ Responses pertaining to the cognitive aspects of social cohesion (eight items) and neighbourhood belonging (seven items) were summed to create the social cohesion subscale (mean = 29.4, SD = 5.5, range 8–40) and neighbourhood belonging subscale (mean = 26.1, SD = 6.0, range 7–35). Neighbourhood quality was assessed using seven questions (mean = 13.3, SD = 3.3, range 7–21), and neighbourhood disorder with seven questions (mean = 7.9, SD = 2.1, range 5–15). Higher scores represented more positive perceptions of the neighbourhood environment.

### Statistical analysis

Propensity scores were estimated using baseline covariates associated with residence in an intervention area and changes in mental health: employment status, housing tenure, council tax band, poverty (defined as earning <60% of the UK median wage at the time of the survey) and marital status. One-to-one matching produced a total analytical sample of 8394 (all 4197 intervention group participants resident in 35 LSOAs were matched to the same number of control group participants, resident in 75 LSOAs). Standardized differences between the intervention and control groups demonstrated a good balance (range = 0.00–0.01). The direct effect of regeneration on changes in mental health was estimated using ordinary least squares regression weighted by resident’s propensity score.^6^ The products of coefficients approach^7^ estimated the association between the residence in an intervention compared with intervention area for: social cohesion, neighbourhood belonging, neighbourhood quality and disorder (the *a* path coefficients). Next, the association between the mediators and mental health (the *b* path coefficients) were estimated. The total indirect effect was the sum of these indirect effects (*a_1_b_1_* + *a_2_b_2_* + *a_3_b_3_* + *a_4_b_4_*).^7^ In order to address the potential for confounding of the mediator/outcome relationship we included the Wave 1 responses for all four mediators and MHI-5 score as suggested by VanderWeele.^8^ In addition, we explored the influence of a more flexible model by incorporating interaction between mediators and saw no substantive differences between the model described earlier (table 1).

**Table 1:** Standardized change and difference in neighbourhood social cohesion, belonging, quality and disorder in control and intervention areas (*n* = 8394) and propensity score weighted standardized coefficients (95% CI) for the indirect, direct and total effect of targeted regeneration on mental health (*n* = 8394)

	Control	Intervention	Difference	*B* (95% CI)
Indirect effect				0.034 (0.021, 0.046)
Social cohesion	0.003 (−0.114, 0.107)	0.004 (−0.043, 0.045)	0.001	0.001 (−0.004, 0.012)
Neighbourhood belonging	−0.031 (−0.082, 0.019)	0.076 (0.044, 0.108)	0.107	0.007 (0.002, 0.020)
Neighbourhood quality	−0.059 (−0.092, −0.026)	0.076 (0.043, 0.108)	0.134	0.014 (0.007, 0.020)
Neighbourhood disorder	−0.056 (−0.090, −0.023)	0.070 (0.038, 0.102)	0.127	0.012 (0.006, 0.018)
Direct effect				0.029 (−0.014, 0.074)
Total effect				0.063 (0.016, 0.110)

### Missing data

All missing values were imputed at baseline and follow-up for all covariates used multiple imputation by chained equations to generate 20 imputed datasets, accounting for the two-level hierarchical structure of the dataset (individuals nested within LSOAs) using the MICE package, v2.30 in R. Analyses were undertaken in Stata 13.1.

## Results

**Figure 1:**
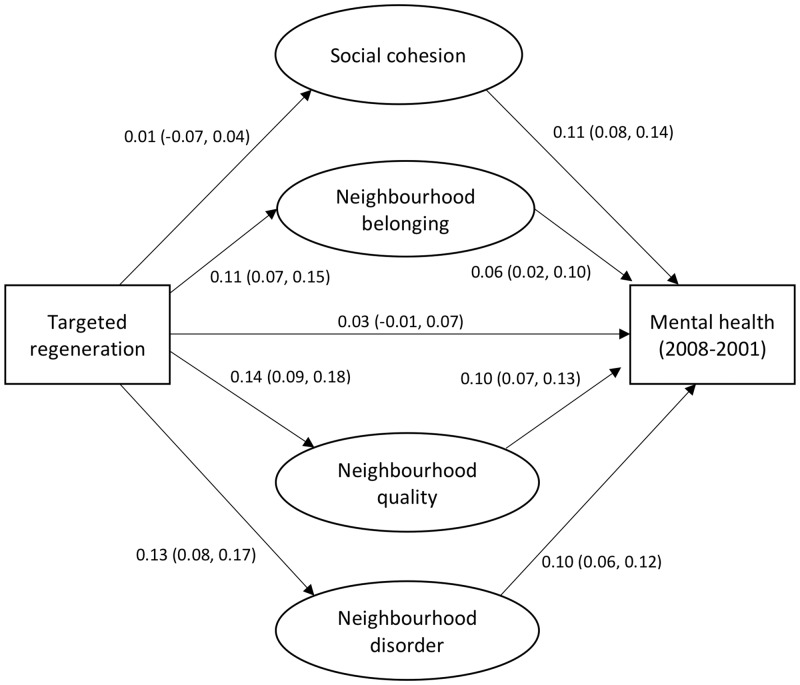
Path diagram of the association between targeted regeneration with social cohesion, neighbourhood-level social cohesion, belonging, quality and disorder in predicting changes in mental health

The 8394 (55.5% women) respondents, mean age was 47.6 (SD = 15.1) demonstrated increased MHI-5 score in the intervention group (1.3, 95% CI = 1.03–2.02) compared with the control group. An initial test for heterogeneity revealed a non-significant interaction between age and gender in MHI-5 scores. The direct effect of regeneration showed improved mental health in residents in the intervention compared with control group (β coefficient = 0.06; 95% CI = 0.02–0.10). Neighbourhood-level mediators explained (0.034/0.063) 54% of the association between regeneration and mental health, social cohesion explained 1.7%, neighbourhood belonging explained 11.1%, neighbourhood quality 22.2% and disorder explained 19% of the total effect (see figure 1 and supplementary table S1).

## Discussion

Targeted regeneration of socioeconomically deprived neighbourhoods was associated with a small improvement in the mental health; thus, inequalities in mental health narrowed. The size of the effect was equivalent to one out of every three intervention group residents increasing their response on the MHI-5 scale by one category (e.g. from ‘most of the time’ to ‘all of the time’) or 7% of a standard deviation on the MHI-5 scale. Over half of this improvement was explained through improvements in neighbourhood belonging, quality and reductions in disorder.

Improved neighbourhood quality explained 22% of the association between regeneration and mental health replicating the findings of an evaluation of regeneration programmes in England and Scotland.^9^^,^^10^ Of the regeneration actives undertaken in Communities First, 22.3% were to improve housing and the physical environment.^2^ We found an association between regeneration and improvement in the perceptions of the quality of their neighbourhood and reductions in disorder. This suggests regeneration activities may have improved features of the physical environment and reduced neighbourhood disorder, rather than changing residents’ perceptions of social cohesion. Therefore, removing graffiti or installing CCTV may illicit a measurable change whereas employing youth centre staff or an arts festival, may be harder to measure. Reducing noise, speeding traffic or providing safe places for children to play in turn reducing symptoms of anxiety.^10^

The strengths of this study are its prospective design with detailed pre- and post-intervention assessments of socioeconomic disadvantage on residents. Propensity scores balance baseline characteristics between intervention and control areas. Generalizability may be limited by an inability to directly capture individual exposure, a common factor in evaluation of neighbourhood-level interventions. This misclassification bias may bias towards the null leading to more conservative estimates. Targeted regeneration, directed by residents of deprived urban communities, aiming to improve the quality of the physical environment and reduce levels of disorder, may help to reduce inequalities in mental health.

## Funding

This project is funded by the National Institute for Social Care and Health Research (NISCHR) programme (project reference RFS-12-05). This study makes use of anonymized data held in the Secure Anonymized Information Linkage (SAIL) system, which is part of the national e-health records research infrastructure for Wales. We would like to acknowledge all the data providers who make anonymized data available for research. This work is undertaken with the support of The Centre for the Development and Evaluation of Complex Interventions for Public Health Improvement (DECIPHer), a UK Clinical Research Collaboration Public Health Research Centre of Excellence. Joint funding (MR/KO232331/1) from the British Heart Foundation, Cancer Research UK, Economic and Social Research Council, Medical Research Council, the Welsh Government and the Wellcome Trust, under the auspices of the UK Clinical Research Collaboration, is gratefully acknowledged. This work is also supported by the Farr Institute of Health Informatics Research. The Farr Institute is supported by a consortium of 10 UK research organizations: Arthritis Research UK, the British Heart Foundation, Cancer Research UK, the Economic and Social Research Council, the Engineering and Physical Sciences Research Council, the Medical Research Council, the National Institute of Health Research, the National Institute for Social Care and Health Research (Welsh Government) and the Chief Scientist Office (Scottish Government Health Directorates).

*Conflicts of interest*: None declared.

**Key points**

- Propensity score matching allows less bias assessment of the effect on mental health of targeted regeneration.
- Targeting regeneration on areas of greater deprivation improves mental health.
- Regeneration narrowed mental health inequalities by improving perceptions of the physical environment and neighbourhood disorder.
- Regeneration was not associated with changes in social cohesion.

## Supplementary Material

ckz221_Supplementary_Data
