# Supplementary material for: Improving mental health through neighbourhood regeneration: the role of cohesion, belonging, quality and disorder
Source: Eur J Public Health. 2019 Dec 10;30(5):964–6. doi: 10.1093/eurpub/ckz221 (PMC7536250; doi:10.1093/eurpub/ckz221)
Supplement: ckz221_Supplementary_Data [file ckz221_supplementary_data.docx]

**Table 1: Standardised change and difference in neighbourhood social cohesion, belonging, quality and disorder in control and intervention areas (n = 8,394) and propensity score weighted standardised coefficients (95% confidence intervals) for the indirect, direct and total effect of targeted regeneration on mental health (n = 8,394)**

|  | **Control** | **Intervention** | **Difference** | ***B* (95% CI)** |
| --- | --- | --- | --- | --- |
| Indirect effect |  |  |  | 0.034 (0.021, 0.046) |
| Social cohesion | 0.003 (-0.114, 0.107) | 0.004 (-0.043, 0.045) | 0.001 | 0.001 (-0.004, 0.012) |
| Neighbourhood belonging | -0.031 (-0.082, 0.019) | 0.076 (0.044, 0.108) | 0.107 | 0.007 (0.002, 0.020) |
| Neighbourhood quality | -0.059 (-0.092, -0.026) | 0.076 (0.043, 0.108) | 0.134 | 0.014 (0.007, 0.020) |
| Neighbourhood disorder | -0.056 (-0.090, -0.023) | 0.070 (0.038, 0.102) | 0.127 | 0.012 (0.006, 0.018) |
| Direct effect |  |  |  | 0.029 (-0.014, 0.074) |
| Total effect |  |  |  | 0.063 (0.016, 0.110) |
